# Effectiveness of core stabilization exercises and routine exercise therapy in management of pain in chronic non-specific low back pain: A randomized controlled clinical trial

**DOI:** 10.12669/pjms.334.12664

**Published:** 2017

**Authors:** Muhammad Waseem Akhtar, Hossein Karimi, Syed Amir Gilani

**Affiliations:** 1 Dr. Muhammad Waseem Akhtar, PT. Doctors Hospital & Medical Center, Lahore, Pakistan; 2 Professor Hossein Karimi, PT. University Institute of Physical Therapy, The University of Lahore, Lahore, Pakistan; 3 Professor Syed Amir Gilani, Allied Health Sciences, The University of Lahore, Lahore, Pakistan

**Keywords:** Exercise Therapy, Low Back Pain, Manual Therapy, Core Stability

## Abstract

**Background & Objective:**

Low back pain is a frequent problem faced by the majority of people at some point in their lifetime. Exercise therapy has been advocated an effective treatment for chronic low back pain. However, there is lack of consensus on the best exercise treatment and numerous studies are underway. Conclusive studies are lacking especially in this part of the world. Thisstudy was designed to compare the effectiveness of specific stabilization exercises with routine physical therapy exerciseprovided in patients with nonspecific chronic mechanical low back pain.

**Methods:**

This is single blinded randomized control trial that was conducted at the department of physical therapy Orthopedic and Spine Institute, Johar Town, Lahore in which 120 subjects with nonspecific chronic low back pain participated. Subjects with the age between 20 to 60 years and primary complaint of chronic low back pain were recruited after giving an informed consent. Participants were randomly assigned to two treatment groups A & B which were treated with core stabilization exercise and routine physical therapy exercise respectively. TENS and ultrasound were given as therapeutic modalities to both treatment groups. Outcomes of the treatment were recorded using Visual Analogue Scale (VAS) pretreatment, at 2^nd^, 4^th^ and 6^th^ week post treatment.

**Results:**

The results of this study illustrate that clinical and therapeutic effects of core stabilization exercise program over the period of six weeks are more effective in terms of reduction in pain, compared to routine physical therapy exercise for similar duration. This study found significant reduction in pain across the two groups at 2^nd^, 4^th^ and 6^th^ week of treatment with p value less than 0.05. There was a mean reduction of 3.08 and 1.71 on VAS across the core stabilization group and routine physical therapy exercise group respectively.

**Conclusion:**

Core stabilization exercise is more effective than routine physical therapy exercise in terms of greater reduction in pain in patients with non-specific low back pain.

## INTRODUCTION

Non-specific low back pain (NSLBP) is described in a recent review of national guidelines as a diagnosis of exclusion, where pain caused by a suspected or confirmed serious pathology or presenting as a radicular syndrome have been ruled out.^1^ The diagnosis of NSLBP is dependent on the clinician being satisfied for not having any specific cause.^2^ The prevalence of LBP in adults has been well documented with a life-time prevalence of over 70%, one year period prevalence of over 50% and a point prevalence of over 20%, although some studies have reported it to be as high as 40%.^3^

The European Guidelines for Management of Chronic NSLBP recommends supervised exercise therapy as a first-line treatment.^4^ Different systematic reviews conducted in past decade have raised a significant concern over the role of exercise in management of low back pain, with scarcity of concrete evidence supporting any specific type of exercise; e.g. flexion / extension biased, strengthening of abdominals, McKenzie, stretching or Williams.^5^-^9^ Clinical guidelines for low back pain recommends remaining active and early return to physical activity as a mean of faster recovery with less concomitant disability.^10^ However, these clinical guidelines are contradictory in practice to prescribing patient specific exercise^11^ that varies according to the individual assessment of the clinician and imply nonspecific general exercise to be prescribed to every low back patient without considering the individual clinical sign.

Exercises for low back pain have evolved over the period of time with specific emphasis on the maintaining the spinal stability.^12^ These types of core stabilization exercises are aimed at improving the neuromuscular control, endurance, strength of muscles central to maintaining dynamic spinal stability. Transversusabdominis (TrA), lumbar multifidi, and other paraspinal, abdominal, diaphragmatic, and pelvic musculature are targeted in core stabilization exercises. Different studies have reported delayed activation of TrA with respect to erector spinae with significant atrophy of multifidus in subjects with chronic low back pain.^13^-^15^ Though there is lack of data regarding the prevalence of different musculoskeletal disorders in Pakistan, low back pain is a significant complaint with which the patients consult their physical therapist or other health care professionals. Since exercise is the main stay of treatment of low back pain prescribed by physical therapist, it is important to determine the type of exercise that is most specific and targeted in management of low back pain. The main objective of this study was to compare the effectiveness of specific stabilization exercises with routine physical therapy provided in patients with nonspecific chronic mechanical low back pain.

## METHODS

This study was a single blind randomized controlled clinical trial conducted at the outpatient department of Orthopedics and Spine Institute, Johar Town, Lahore. This study was completed in a time period of two years after the approval of synopsis form Institutional Review Board and Ethical Review Committee of University Institute of Physical Therapy, University of Lahore.

### Subject Selection & Sampling Procedure

A pilot study was conducted prior to this study to see the efficacy of core stabilization exercise and routine physical therapy exercise in low back pain patients. Based on the results of the pilot study using a formula as described by Sakpal,^16^ estimated on the basis of pain measured on visual analogue scale at six week follow-up and assuming 80% power, 5% of significance, and 10% drop out rate, to detect a clinically meaningful difference between groups of two scores on a visual analogue scale, a minimum total sample size of 100 was required for the study. The sample was recruited using a non-probability sampling technique. Patients with nonspecific chronic mechanical low back pain, with age between 20-60 years, both male and female gender were included in the study. Patients with disc pathology and radicular pain, acute low back pain, history of spinal fracture or spinal surgery, spondylolisthesis, any systemic disease or TB of spine were excluded from the study. Subjects that had previously received physical therapy treatment for low back pain in a period of six months were also excluded from the study.

### Visual Analogue Scale

Intensity of pain was evaluated with Visual Analogue Scale (VAS) which is reliable and valid measure of pain intensity and it is sensitive to clinical changes in pain.^17^ A zero at left end of the scale indicates no pain while 10 indicates a worst imaginable pain. A change of 1.1-1.2 cm indicates a minimal improvement, which is clinically significant.^18^

### Treatment Groups

***Core Stabilization Exercise Group:*** Subjects allocated to this group were managed with core stabilization exercise targeting deep muscles of the abdomen. This consisted of battery of exercises (Table-I) that are explained by Kisner^19^ along with a baseline therapeutic treatment of ultrasound and TENS. These exercises were supervised by physical therapist.

**Table-I:** List of exercises performed under core stabilization and routine physical therapy exercise.

*Core Stabilization Exercises*	*Routine Physical Therapy Exercises*
1.Pressure feedback core exercise in supine & prone	1.Hamstring stretching
2.Multifidus exercise	2.Calf stretching
3.Frontal & Side Plank exercise	3.Hip flexors stretching
4.Pelvic floor exercises	4.Back extensors stretching
5.Wobble board oblique twist	5.Abdominal curl-up exercise in supine
6.Thera-band reverse wood chop exercise	6.Back extensors exercise in prone
7.Windshield wiper exercises	7.Hip extensors exercises in prone
8.Diaphragmatic strengthening exercises
9.Single leg standing on foam
10.Tandem standing with perturbation in form of rapid arm movements

***Routine Physical Therapy Exercise Group:*** Subjects allocated to this group were managed with routine physical therapy exercise that were not specifically targeted to core muscle of the spine along with a baseline therapeutic treatment of ultrasound and TENS (Table-I). These exercises were supervised by physical therapist.

**Fig. 1:**
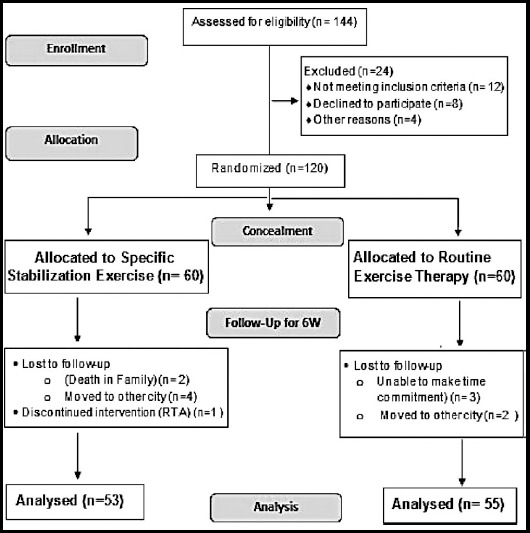
Flow Sheet Diagram of the Research Process.

***Data Collection Method:*** Patient’s basic demographic history and contact details was taken after their signed consent. A detailed musculoskeletal examination of lumbar spine was performed before the start of treatment. Pretreatment reading for pain was noted. Subjects were randomly allocated to two treatment groups A & B using computer generated random number table and were blinded form the treatment they received. An expert physical therapist with more than 10 years clinical experience helped the patients in performing either core stabilization exercise (Group-A) or routine physical therapy exercise (Group-B). The session with the physical therapist usually lasted for up to 40 minutes with 5-10 minutes rest interval. All the subjects in both groups were treated with one treatment session per week for up to six weeks. Patients were reminded for their routine appointment one-day prior using telephonic call. Post treatment readings of pain were recorded at end of 2^nd^, 4^th^ and 6^th^ treatment week (Fig. 1). All the subjects were managed with the base line treatment of therapeutic ultrasound (3MH for ten minutes at 50% intensity) and TENS (Continuous mode for 10 minutes) at lumbar spine as a baseline treatment in both groups. Furthermore, all the patients were also instructed to do same exercise twice a week at home by the help of printed handouts given by the physical therapist and were asked to refrain from heavy intensity physical work during the course of treatment.

### Data Analysis

The data was analyzed using SPSS for Windows software, version 20. Statistical significance was set at *P* = 0.05. Frequency tables were used to show summary of group measurements measured over time. Wilcoxon t test was used to show the progress of two groups between two successive visits. Mann Whitney U test and Friedman ANOVA was used to show change in pain score across and within each group respectively.

## RESULTS

Comparison of the socio-demographic profile of the subjects that participated in the study is illustrated in Table-II. The subjects in both treatment groups were comparable at the baseline. Age of the participants varied form 24 – 59 years across both groups. The mean age of participants was 46.39 ± 7.43years in core stabilization exercise group as compared to 45.50 ± 6.61years in routine physical therapy exercise group.

**Table-II:** Comparison of Socio-demographic data of the 2 treatment groups.

*Demographic Variables*	*Core Stabilization Exercise Group (n= 53)*	*Routine Physical Exercise Group (n= 55)*	*P value*
Age (Years ± SD)	46.39 ± 7.43	45.50 ± 6.61	0.09
Height (m ± SD)	1.62 ± 0.08	1.60 ± 0.08	0.04
Weight (kg ± SD)	64.03 ± 10.00	63.69 ± 9.15	0.45
BMI (kg/m2 ± SD)	24.15 ± 2.38	24.82 ± 3.02	0.10

**Table-III:** Comparison of base line and final value for VAS across 2 treatment groups with their mean difference & P value

*Measure*	*Group*	*Baseline*	*Final*	*Mean Change*	*P value*
VAS	Core Stabilization EX	5.77 ± 1.08	2.69 ± 0.93	3.08	<0.01
	Routine Physical Therapy Ex	5.40 ± 1.24	3.69 ± 0.79	1.71	<0.01

This study found statistically significant difference in pain across the two groups at 2^nd^, 4^th^ and 6^th^ week of treatment with p value less than 0.05. The mean reduction of pain was 3.08 and 1.71 across the core stabilization group and routine physical therapy exercise group respectively.

## DISCUSSION

Results of this study showed that both exercise proved to be effective in management of low back pain statistically but clinically there was greater pain reduction in core stabilization exercise group as compared to routine physical therapy exercise group. A pilot randomized controlled trail conducted by Areeudomwong et al. measured the effect of 10 weeks core stabilization program on pain presentation pattern, disability and activation of trunk muscles in subjects with clinical instability of the lumbar spine. The subjects in the control group were treated with stretching of the trunk muscles and hydro collator therapy. Results of their study indicated decreases in pain and disability in both treatment groups similar to the findings of this study. However, the improvement in the activation ratio of transversusabdominus and internal oblique relative to rectus abdominus muscle was found in the subjects that were treated with core stabilization exercise.^20^ It has been hypothesized that core stabilization exercise enhance the ability of the segmental muscles that result in improved function and decreased pain in subject with chronic nonspecific low back pain.

Subjects allocated to core stabilization group demonstrated a decrease in pain. These findings were also reported in similar studies by Koumantakis and O’Sullivan of chronic low back pain,^21^ spondylolysis or spondylolisthesis.^22^ O’Sullivan^22^ signified that abdominal drawing in maneuver (ADIM) helps in integration of muscles into a task by providing a powerful biofeedback. Similar findings were also reported in another study in which ADIM aided in stabilization of the lumbar spinal segments during functional task performing in healthy subjects.^23^ In this study core stabilization exercises also significantly reduced pain in subjects with low back pain.

A study conducted by Costa et al.^24^ also established the superiority of motor control exercises over electrotherapeutic modalities used to treat chronic nonspecific low back pain. Subjects in treatment group were treated specific exercises targeting the activation of the transversusabdominus and multifidus. When appropriate control was developed subjects were progressed to more complex functional task targeting the activation of the core muscles. Control group was treated with detuned short wave diathermy and placebo ultrasound therapy for 20 minutes over 8 treatment session for 12 week. Results showed significant reduction in pain measured on NPRS and disability measured on Roland-Morris Disability Questionnaire across the two groups but this reduction was clinically more significant in treatment group compared to control group.

## CONCLUSION

Core stabilization exercise is more effective than routine physical therapy exercise in terms of greater reduction in pain in chronic nonspecific low back pain.
